# Ultralow Limit Detection of Soluble HER2 Biomarker in Serum with a Fiber-Optic Ball-Tip Resonator Assisted by a Tilted FBG

**DOI:** 10.1021/acsmeasuresciau.2c00008

**Published:** 2022-03-15

**Authors:** Marzhan Sypabekova, Aida Amantayeva, Luca Vangelista, Álvaro González-Vila, Christophe Caucheteur, Daniele Tosi

**Affiliations:** † Nazarbayev University School of Medicine, 53 Kabanbay Batyr Avenue, 010000 Nur-Sultan, Kazakhstan; ‡ Nazarbayev University School of Engineering and Digital Sciences, 53 Kabanbay Batyr Avenue, 010000 Nur-Sultan, Kazakhstan; § Baylor Research and Innovative Collaborative, Baylor University, 100 Research Pkwy, Waco, Texas 76704, United States; ∥ Electromagnetism and Telecommunication Department, University of Mons, Boulevard Dolez 31, 7000 Mons, Belgium; ⊥ National Laboratory Astana, Laboratory of Biosensors and Bioinstruments, 010000 Nur-Sultan, Kazakhstan

**Keywords:** optical-fiber biosensors, fiber Bragg grating, low-limit biosensor, human epidermal growth factor-2 (HER2), HER2 detection, cancer biomarker detection, cancer diagnostic

## Abstract

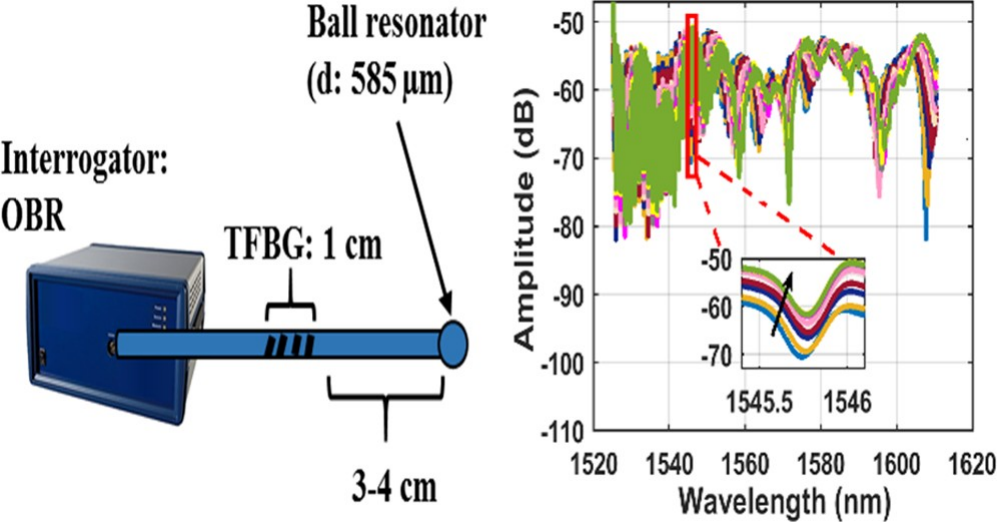

An optical-fiber biosensor has been developed for the detection of the breast cancer biomarker soluble human epidermal growth factor receptor-2 (sHER2). The sensor was fabricated by combining a tilted fiber Bragg grating (TFBG) with a ball resonator, allowing us to achieve an excellent sensitivity compared to other optical-fiber-based sensors. The sensor exhibits a resonance comb excited by the TFBG and the spectral profile of the ball resonator. The detection of sHER2 at extremely low concentrations was carried out by tracking the amplitude change of selected resonances. The therapeutic anti-HER2 monoclonal antibody Trastuzumab has been used to functionalize the biosensor with silane surface chemistry. The sensor features a sensitivity of 4034 dB/RIU with a limit of detection (LoD) in buffer and in a 1/10 diluted serum of 151.5 ag/mL and 3.7 pg/mL, respectively. At relatively high protein concentrations (64 ng/mL) binding to sHER (7.36 dB) as compared to control proteins (below 0.7 dB) attested the high specificity of sHER2 detection.

## 1 Introduction

Early detection of cancer is an essential aspect to warrant the most efficacious prognosis and intervention. Equally important to early-stage tumor detection is the diagnosis of tumor relapse following treatment, an area where a protein biosensor would also be most relevant. Abnormal levels of cancer biomarkers in physiological fluids, for instance, blood, most likely indicate the occurrence of a disease, directing to the need for more precise studies for the determination of the type of cancer and its development stage. An important example of such biomarkers is human epidermal growth factor-2 (HER2), which belongs to the family of epidermal growth factor receptors. Overexpression of this protein is usually associated with the occurrence and progression of aggressive types of breast cancer. The detectable range for soluble HER2 in the serum of healthy individuals is 2–15 ng/mL and that in breast cancer patients is 15–75 ng/mL.^1,2^ Elevated levels of soluble HER2 in the saliva of patients with breast carcinoma, as compared to healthy individuals, have also been reported.^3,4^ HER2 is a transmembrane protein, and the extracellular domain is often cleaved by proteolytic shedding into the blood circulation, leaving a constitutively active truncated receptor on the cell membrane.^5^ HER2 shedding is a common feature in tumor metastasis and the truncated receptor has a superior oncogenic activity, promoting cell proliferation and survival. Therefore, detection of soluble HER2 (sHER2) is particularly relevant for the diagnosis of cancer recurrence and metastasis. Moreover, overexpression of HER2 has also been associated with the resistance to certain chemotherapeutics,^6^ risk of brain metastasis,^7^ and other types of cancers such as stomach, ovarian, and lung cancer.^8^ In blood, sHER2 can be detected in approximately 45% of patients with HER2-related breast cancers.^9^

Rapid and label-free sHER2 detection has been recently investigated using surface plasmon resonance, piezoelectric microcantilever, electrochemical, optofluidic ring resonator, and surface acoustic wave biosensors.^10−15^ However, these techniques are limited in one or several factors, such as high cost and long testing time, complicated fabrication steps, and limited performance in sHER2 detection in biological fluids. Unlike the above biosensor types, optical-fiber-based biosensors have significant advantages including cost-effectiveness, small size, flexibility, lightweight, remote detection capability,^16^ minimal invasiveness, and immediate detection in nonliquid media such as human tissues.^17,18^ The interest in optical-fiber biosensors arises from their medical applicability in the field of endoscopy and laser surgery.^19^ Studies have shown that it is also possible to detect biological binding processes occurring around the fiber by modifying the fiber structure (grating inscription, nanoparticle deposition, etching, tapering). The physical characteristics of the optical fiber allow it to be inserted inside the needle or a catheter, making it prone to be designed as a hand-held probe for in situ measurements. In addition, optical-fiber-based biosensors are biocompatible (according to the ISO 10993 standard), capable of working in hazardous environments and complex biological media,^17^ and immune to electromagnetic interferences, allowing the sensor to operate in magnetic resonance imaging environments.

In this work, we demonstrate the use of a tilted fiber Bragg grating (TFBG) together with a ball resonator for the detection of sHER2. The TFBG consists of a periodic and localized refractive index modulation of the core of a single-mode optical fiber that is angled with respect to the fiber axis, reflecting certain light modes while transmitting all others. As some modes are reflected toward the cladding, the surrounding of an optical fiber containing a TFBG turns into a suitable platform for biological detection. The biochemical events occurring on the surface of such fiber can be monitored through the tracking of the spectral changes experienced by those modes and providing a limit of detection (LoD) at the pM level.^20^ A ball resonator is a spherical device fabricated at the tip of single-mode glass fiber with a ball diameter in the range from 300 to 650 μm. As shown in previous works,^21,22^ the ball resonator is a weakly reflective device, characterized by a quasi-random broadband spectrum and a high sensitivity to the refractive index. The change in the surrounding medium (in the form of binding between the analyte and the receptor) in acting upon the evanescent wave can be detected in the form of a wavelength shift and an amplitude change. Ball resonators can be fabricated with a CO_2_ laser splicer in less than one minute with simple preparation and provided an LoD at pM level and had the sensitivity of 1273.74 nm/RIU.^21^ In this work, we combined TFBG with a ball resonator, which showed an increased sensitivity in buffer (LoD at an aM level) with respect to the TFBG or ball resonator alone (LoD at a pM level). Additionally, since both devices work on telecom-grade single-mode fibers, it is possible to exploit the extremely high accuracy in wavelength and amplitude detection given by infrared interrogators, such as an optical backscatter reflectometer (OBR).

The capture of sHER2 was carried out on the surface of the TFBG–ball resonator where Trastuzumab was used as a receptor. Trastuzumab is a therapeutic anti-HER2 humanized monoclonal antibody (mAb) widely used in the clinical diagnosis and treatment of breast cancer.^23^ The TFBG–ball resonator configuration allowed to achieve a sensitivity of 4034 dB/RIU (refractive index unit). In this work, a TFBG with a tilt angle of 5° was spliced in close proximity to the ball resonator and different concentrations of sHER were detected both in buffer and serum, presenting a limit of detection at much lower concentrations than those previously reported for sHER2 optical-fiber sensors. Ultralow detection of HER2 in serum by this novel biosensor design represents an encouraging technological advance prone to its implementation in the early diagnosis and relapse of breast cancer.

## 2 Materials and Methods

### 2.1 Materials

(3-Aminopropyl) trimethoxysilane (APTMS), glutaraldehyde, phosphate-buffered saline (PBS), thrombin protein, hydrogen peroxide (H_2_O_2_), methanol, human serum, and sulfuric acid (H_2_SO_4_) were purchased from Sigma-Aldrich (Darmstadt, Germany). d-Sucrose and bovine serum albumin (BSA) were obtained from Thermo Fisher Scientific (Runcorn, U.K.). Absolute ethanol was purchased from Aidabul Distillery (Kokshetau, Kazakhstan). Protein solutions were prepared freshly each time in PBS at pH 7.4. Human ErbB2/HER2 (Research Grade Trastuzumab Biosimilar) antibody and recombinant human ErbB2/HER2 protein were purchased from BEI Resources (Manassas). IL4 and CCL5 were purchased from Gibco (Massachusetts). All aqueous solutions were prepared using 18.2 MΩcm ultrapure water with a Pyrogard filter (Millipore, U.K.).

### 2.2 Fabrication of the Optical-Fiber Biosensor

The TFBGs used in this work are similar to the ones originally used in ref (24). A Noria FBG manufacturing system from NorthLab Photonics was used for FBG fabrication in a hydrogen-loaded photosensitive single-mode optical fiber (PS1250, Fibercore U.K.). This tool integrates an excimer ArF laser emitting at 193 nm and a set of phase masks, so (T)FBGs with different features can be fabricated by minimal modifications on its settings. In this case, a phase mask with a grating pitch of 1078 nm was chosen. This phase mask has the grating holographic pattern that is already tilted so that TFBGs with a tilt angle of 10° are inherently produced without any additional consideration. Several 1 cm long TFBGs were fabricated for the experiments. To ensure an optimal spectral response, the laser energy was set to 5 mJ and the repetition rate to 50 Hz. Finally, the photoinscription process took place through three bursts of 7500 pulses for each TFBG.

Ball resonators were fabricated in standard single-mode fiber using a CO_2_ laser splicer (LZM-100 CO2, Fujikura Ltd., Japan) as described in ref (21). This method allows rapid fabrication of the fiber-tip sphere through a popular method used in diffractive lens manufacturing. A ball resonator with a diameter of 585 μm was used for functionalization.

The TFBG–ball resonator incorporates the two elements, both sensitive to the outer refractive index (RI). The TFBG is characterized by a comb of RI-sensitive cladding mode resonances, which are visible on the interrogator at the lower-wavelength region. The ball resonator shows as a low-finesse spectrum resembling a weak interferometer. When the TFBG and ball resonator are spliced together, the resulting spectrum forms a comb of cladding modes exhibiting a higher intensity change. This results in a higher peak-to-peak intensity mainly observed in the leftmost portion of the spectrum. Since the TFBG is a transmission device, the addition of a ball resonator at the fiber tip allows us to combine its transmission–reflection spectrum^25^ with the ball resonator reflection spectrum.

**Figure 1:**
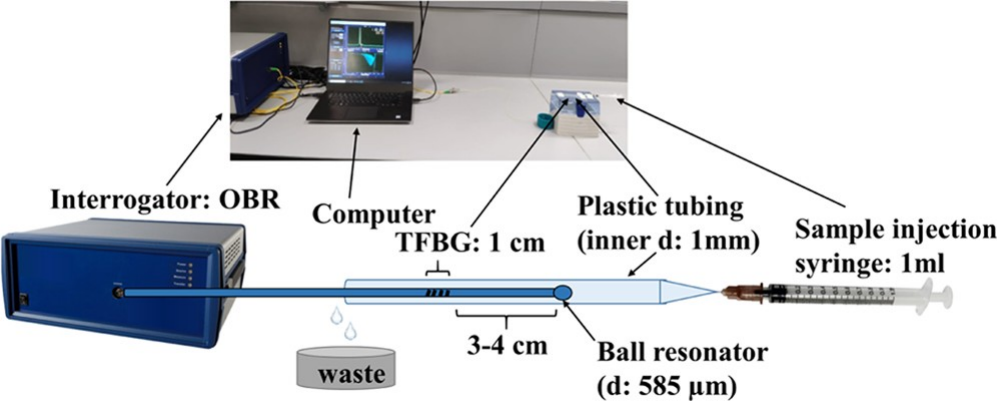
Experimental setup.

Optical fibers containing TFBGs and ball resonators, respectively, were cleaved at one end using a cleaver (CT30 Fujikura Ltd., Japan). Next, the cleaved ends were spliced together using an optical-fiber splicer (36S Fujikura Ltd., Japan). The region containing the TFBG–ball resonator structure was further functionalized with antibodies. After biofunctionalization, the TFBG–ball resonator was placed inside an 11 cm long silicone tubing with an inner diameter of 0.9 mm, as illustrated in Figure 1. The injection of solutions was done with a 1 mL syringe at one end of the tubing, and the flow-through was collected in a waste container at the other end.

### 2.3 Instrumentation and Experimental Setup

The reflection spectra of the TFBG–ball resonator were measured using an Optical Backscatter Reflectometer (OBR 4600, Luna Inc.) with a resolution of 8 pm, on a wavelength window between 1530 and 1616.4 nm. The integration range was set to 20 cm on the OBR instrument. The interrogator was used to collect the TFBG–ball resonator spectra throughout calibration, fabrication, and functionalization steps, as well as during the protein detection measurements. The experimental setup is shown in Figure 1. The amplitude change of the most sensitive mode was chosen to report the results.

An interrogator was directly connected to the TFBG–ball resonator. The TFBG–ball resonator part was embedded in the tubing. The solution inside the tubing was injected using a syringe and the waste was collected in a separate container.

### 2.4 Refractive Index (RI) Calibration

Solutions with different RIs were prepared by dissolving different amounts of sucrose in water. The RI of each solution was measured using an automatic digital refractometer (Anton Paar, Inc., Abbemat 300). The RI range was between 1.34722 and 1.34873. The calibration with varying RI was performed by letting 500 μL of each sucrose solution flow inside the silicone tubing with a 1 mL syringe.

### 2.5 Biofunctionalization and Protein Detection

**Figure 2:**
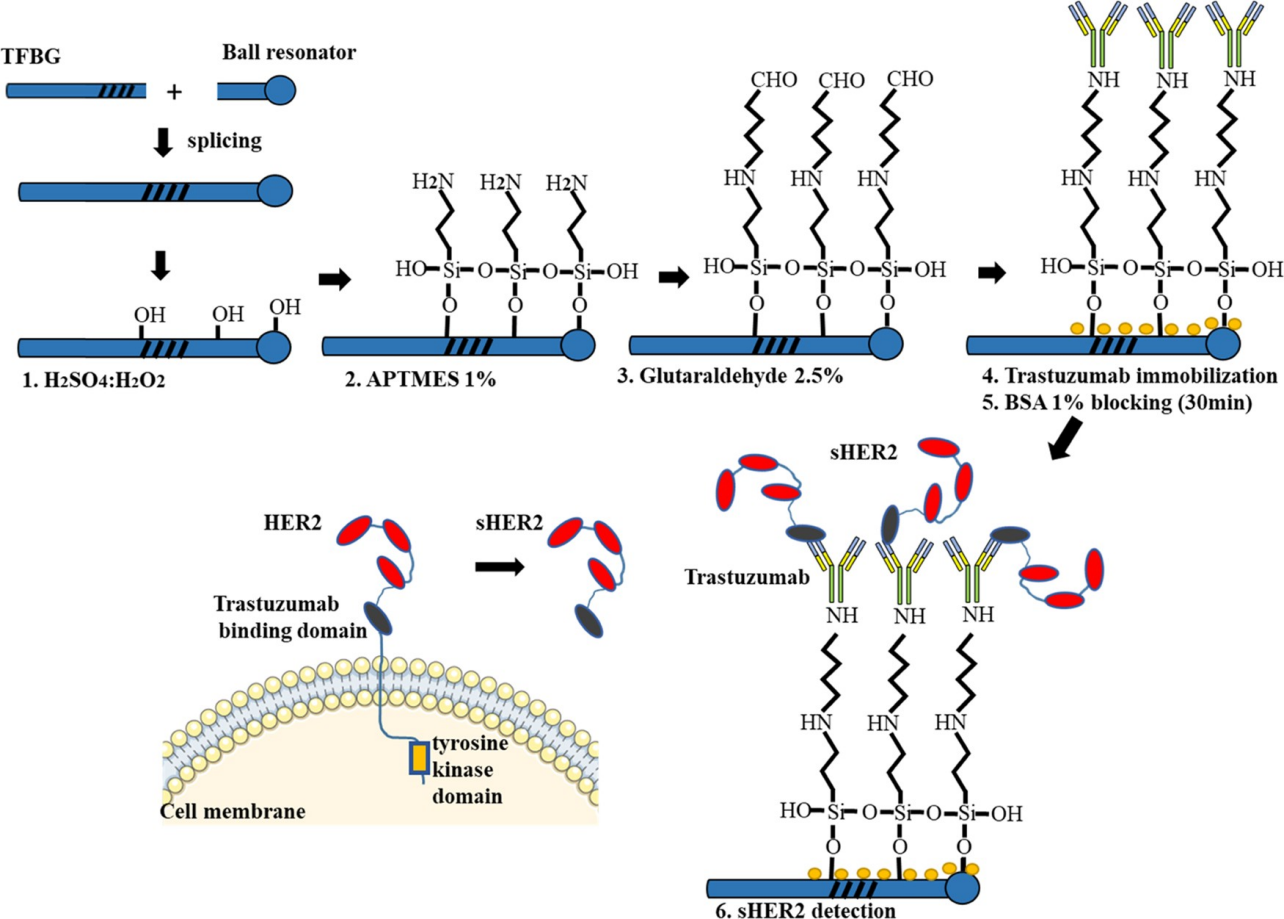
Surface modification steps and molecular basis of the sHER2 system.

The surface of the TFBG–ball resonator fiber (made of glass) was first cleaned with a Piranha solution (3:1 v/v H_2_SO_4_/H_2_O_2_) for 10 min, followed by thorough rinsing with H_2_O. The surface of the clean fiber was then covered with aminosilanes as the result of the silanization process. The salinization process was carried out by immersing the TFBG–ball resonator fiber in 1% (3-aminopropyl) trimethoxysilane (APTMS) diluted in methanol for 20 min at room temperature (controlled to ±1 °C throughout experiments). This resulted in the formation of a covalent −Si–O–Si– bond between the glass and aminosilanes. The fiber was then rinsed with methanol to remove unreacted aminosilanes and air-dried. The fiber was then placed in an oven at 80% for 30 min. The silanized fiber was then placed immersed in 2.5% glutaraldehyde solution for 1.5 h at room temperature in PBS (pH 7.4) on a rotary shaker at 60 rpm. The fiber was then thoroughly rinsed with PBS and immersed in anti-HER2 antibodies solution at 20 μg/mL in PBS^30^ at 4 °C overnight. The unbound antibodies were washed by rinsing with PBS and the unreacted surface was blocked by incubating with 1% BSA for 30 min. The fiber surface was then rinsed with PBS, followed by protein incubations. Detection of the target sHER2 was achieved by incubating the sHER2 protein for 15 min at room temperature on the surface of the TFBG–ball resonator that was pre-immobilized with antibody. For the detection studies, a wide range of sHER2 concentrations was used from 3 ag/mL up to 128 ng/mL and the sHER2 protein was diluted in PBS. For specificity studies, different 64 ng/mL of the target sHER2 and nontarget proteins IL4, CCL5 and thrombin diluted in PBS buffer were incubated for 15 min on the fiber surface. After the incubation, the fiber surface was rinsed with PBS to remove the unbound proteins. The signal recording was conducted in PBS. The schematics of the biofunctionalization step are shown in Figure 2.

### 2.6 Detection of sHER2 in Serum

A 1/10 serum dilution was made in the PBS buffer. The antibody-functionalized TFBG–ball resonator surface was first stabilized in diluted serum until a stable signal was obtained from the optical measurement. The fibers were then incubated in diluted serum with a wide range of sHER2 protein concentrations for 15 min at room temperature. After the incubation, the fiber surface was rinsed with PBS to remove the unbound proteins. The signal recording was conducted in PBS.

TFBG and ball resonators were spliced together and used as a sensing surface. The sensing surface was functionalized with the Trastuzumab antibody and cross-linked via glutaraldehyde. The unreacted surface was blocked with BSA molecules. The sensor was used to detect the sHER protein. HER2 is a transmembrane protein, and the extracellular domain is often cleaved by proteolytic shedding into the blood circulation. The sHER protein has a domain that is specific to the Trastuzumab antibody.

### 2.7 Signal Analysis

The interrogation was performed with OBR 4600. Return loss (RL) and wavelength data were processed in MATLAB. To reduce the noise of the obtained signal, the low-pass filter was implemented (Butterworth, fifth order, with 0.015 digital frequency cutoff.). For RL versus concentration graphs, the reference was scanned before the experiments. Amplitude change was calculated by subtracting reference (measurement in PBS) from concentrations. Also, the second-order polynomial fit of log 10 of the concentration was implemented for sHER2 graphs, and their coefficients of determination were calculated.

Spectral changes have been measured by selecting the most sensitive cladding mode, for each type of detection, along the low-wavelength part of the spectrum, within a 1525–1546 nm window. The response was measured by selecting the most suitable spectral dip and tracking its reflectivity level using a local minima function.^26^ Through this method, the amplitude (or return loss) difference Δ*A* from the reference condition (in PBS) was measured. Amplitude tracking of a selected cladding mode allows us to compare the results to plasmonic TFBG devices, as reported by Caucheteur et al.^27^ As shown in ref (21), spectral variations can be observed throughout the characterization process due to the effects of the ball resonator. It is possible to obtain a significant pattern for spectral variations by tracking the most sensitive cladding mode within the sensitive wavelength range.

Limit of detection (LoD) was found for small sHER2 concentrations using a method reported by Chiavaioli et al.^28^ for grating-based biosensors. The data were fitted using the equation LoD = *f*^–1^(*y*_blank_ + 3σ_max_) for the amplitude change analysis; where *y*_blank_ is the response at the lowest sHER2, σ_max_ is the maximum of the standard deviation (0.198 dB) observed through the measurement, and *f* is the fitting curve. The standard error was found to be 0.0626 dB.

## 3 Results and Discussion

### 3.1 Refractive Index (RI) Calibration

**Figure 3:**
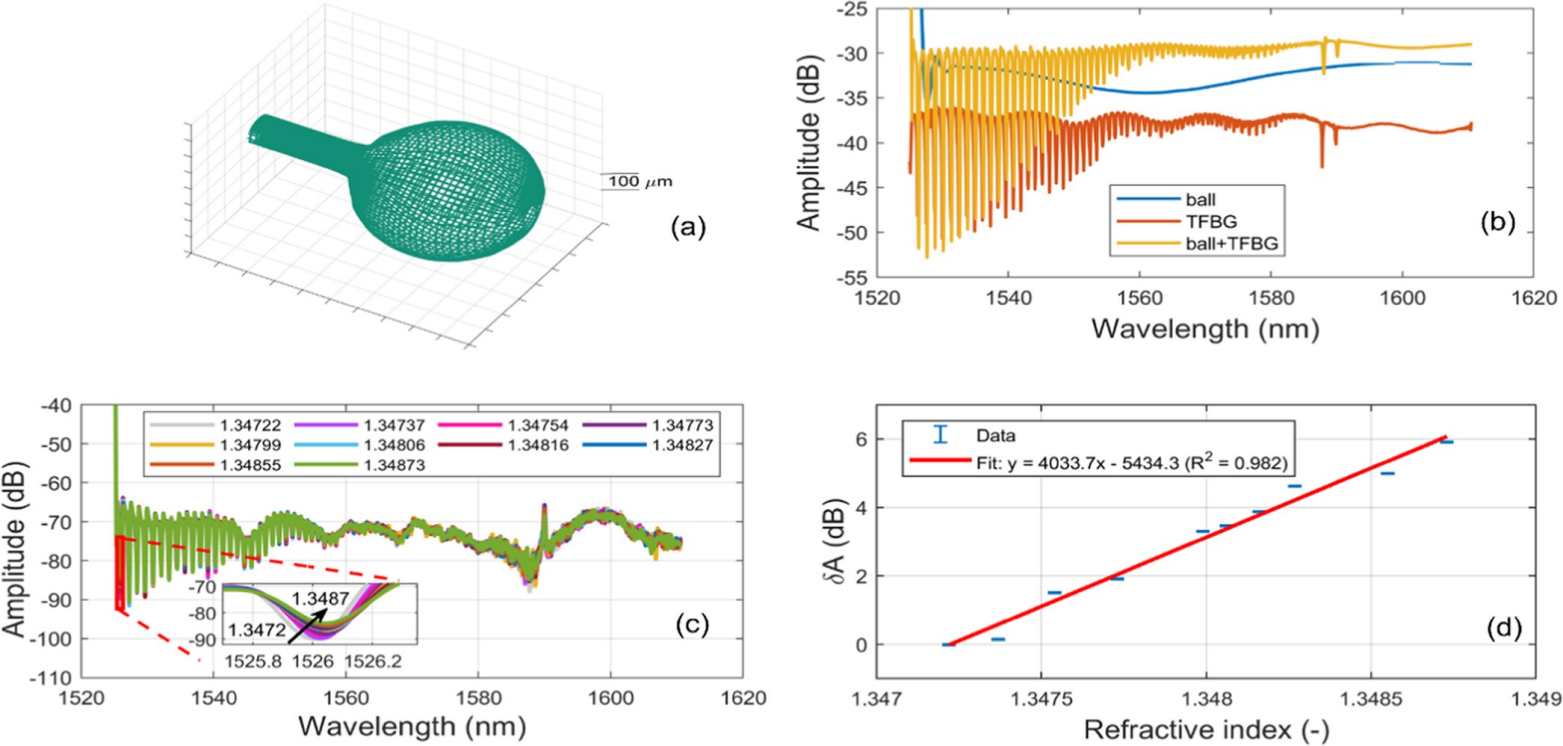
(a) Ball resonator mesh built based on ball fabrication profile; *d* = 585 μm, measured from the profilometry function of the splicing machine. (b) Reflection spectra of the TFBG, ball resonator, and TFBG–ball resonator in the air. (c) Spectra of TFBG–ball resonator at different RI concentrations illustrating the change of amplitude. The inset shows the tracked cladding mode dip around 1526 nm. (d) Amplitude change versus RI change evaluated on all values of RI. The sensitivity has been estimated with a linear fit. The standard error is 0.02 dB, which is the uncertainty of the OBR instrument.

The constructed ball profilometry is illustrated in Figure 3a. The spectrum of TFBG, ball resonator, and TFBG–ball resonator in the air is illustrated in Figure 3b. The TFBG–ball resonator was calibrated in different sucrose solutions with different RI values from 1.34722 to 1.34873 RIU. The calibration was conducted to establish the sensitivity of the device to the changing surrounding medium. The overlay spectra at different RIs are presented in Figure 3c. The most sensitive mode with the biggest amplitude change was further chosen for data analysis. The inset graph depicts that with the increase of RI value, the amplitude of the return loss increases. The amplitude change was linear with a sensitivity of 4034 dB/RIU and the coefficient of determination was 98.2% (Figure 3d). The sensitivities of other optical-fiber-based sensors previously developed for sHER2 detection mainly focused on wavelength change (nm/RIU). Previously, gold-coated TFBG sensors for the detection of a different biomolecule (cytokeratin 7) showed a sensitivity of 693 dB/RIU^27^ in different RIs. Indeed, the combination of TFBG and ball resonator increases the overall sensitivity of the device as compared to the sensors where TFBG and ball resonators were used separately (Table 1). The device was further tested for the detection of sHER both in PBS and in a 1/10 diluted serum.

**Table 1:** Comparison of Previously Reported Biosensors Based on Optical Fibers for sHER2 Detection

optical fiber type	receptor type (s)	LOD in buffer	sensitivity	LOD in serum	refs
multimode fiber	anti-HER2 ssDNA aptamers, anti-HER2 antibodies as signal enhancers	with signal enhancer 9.3 ng/mL	808–1650 nm/RIU	unknown	(30)
TFBG	anti-HER2 ssDNA aptamers; anti-HER2 antibodies	unknown	unknown	unknown	(31)
tapered fiber	HER2 antibody	0.1 ng/mL	unknown	2 ng/mL	(32)
tapered fiber and optical-fiber ring resonator	HER2 antibody		30 nm/RIU	10 ng/mL	(14)
tapered multimode fiber	HER2 antibody	50 ng/mL	6878 nm/RIU	unknown	(33)
tapered FBG	HER2 antibody	2 ng/mL	2333 nm/RIU	unknown	(34)
TFBG	anti-HER2 ssDNA aptamers	10 pg/mL	125 nm/RIU	unknown	(20)
TFBG–ball resonator	HER2 antibody (Trastuzumab biosimilar)	151.5 ag/mL	4034 dB/RIU	3.7 pg/mL	this work

### 3.2 Detection of sHER

**Figure 4:**
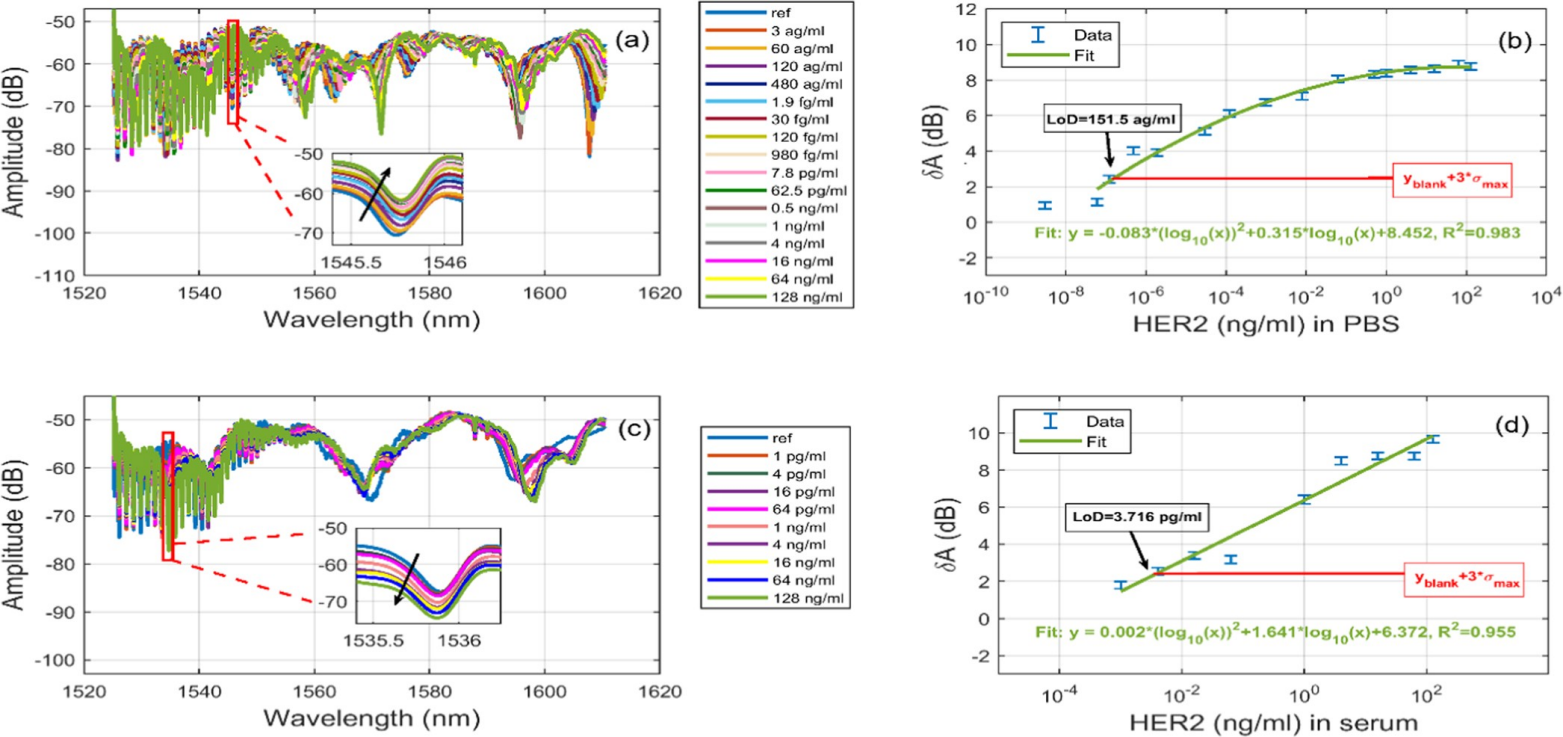
(a) Spectrum after incubating with different sHER2 concentrations in PBS and (c) serum. The insets show amplitudes in the range where the biosensor had the biggest response and provide information regarding the direction of amplitude change. (b, d) Amplitude change versus sHER2 concentrations in PBS and in a 1/10 diluted serum, respectively. *y*_blank_ is the response at the lowest sHER2, σ_max_ is the maximum of the standard deviation (0.198 dB) observed through the measurement, and *f* is the fitting curve. The standard error was found to be 0.0626 dB.

To establish the sensor performance, different sHER concentrations were tested. Before the protein incubations, the functionalized TFBG–ball resonator was stabilized in PBS. The stabilization step was important to ensure the integrity of the sensor surface as well as the maintenance of a stable signal before protein detection. Different sHER concentrations from 3 ag/mL to 128 ng/mL were tested for binding onto the functionalized fiber surface. After protein incubations, the sensor surface was rinsed with PBS to remove unbound molecules and the signal was recorded in the same buffer. As reported in Figure 4a, there is an upward trend of amplitude change at different sHER concentrations. The selected mode for the analysis (most sensitive) was between 1545.5 and 1546 nm. The amplitude rose as the concentrations of sHER increased (Figure 4b). The sensitivity was up to 0.0647 dB per each 10× increase in concentration and the coefficient of determination was 98.3%. The obtained LoD in buffer was 151.5 ag/mL. At lower concentrations (below 60 ag/mL), the sensor did not respond well. The signal started to increase above 60 ag/mL.

### 3.3 Detection of sHER in Serum

The detection of sHER in serum was conducted after spiking the TFBG–ball resonator surface with a 1/10 diluted serum in PBS. The measurement with the protein was conducted only after obtaining a stable signal as the result of consecutive measurements. Different concentrations of sHER were prepared by dissolving sHER in a 1/10 diluted serum. After incubation with sHER, the surface of the fiber was rinsed with PBS and the measurement was conducted in PBS. Interestingly, in the serum, the amplitude change had a downward trend as shown in Figure 4c,d. The selected curve fitting gave the value *R*_2_ = 0.96, showing a relatively high correlation. Similar curve fitting was used in ref (29), where there is a decrease in sensitivity at the higher concentrations. The chosen mode for serum measurements was between 1535.5 and 1536 nm. The sensitivity was up to 0.867 dB per each 10× increase in concentration and the coefficient of determination was 95.8%. The obtained LoD of the sensor in diluted serum was 3.7 pg/mL, which is far below the current clinical detectable range for sHER (15–75 ng/mL for breast cancer patients).^1,2^ The difference between the LoD in buffer and diluted serum suggests that some of the serum components (could be endogenous sHER2) are likely to interfere with the sensor surface. Therefore, to differentiate the interference signal, the fiber was first stabilized in diluted serum, after which the known sHER concentrations in diluted serum were measured. The high sensitivity of the current biosensor in a 1/10 diluted serum increases the possibility of using a small serum sample and dilutes it to a level at which normal individual sHER2 may not be detected and cancer patients could be discriminated for the residual presence of sHER2. This could possibly further lower the limit of detection (turn the PBS measurements to a practical diagnostic advantage), leading to a more accurate (and most likely more reproducible) detection. The current sensor can be further tested in diluted human serum from patients as well as compare the performance with those used in the clinic.

### 3.4 Specificity of the TFBG–Ball Resonator

**Figure 5:**
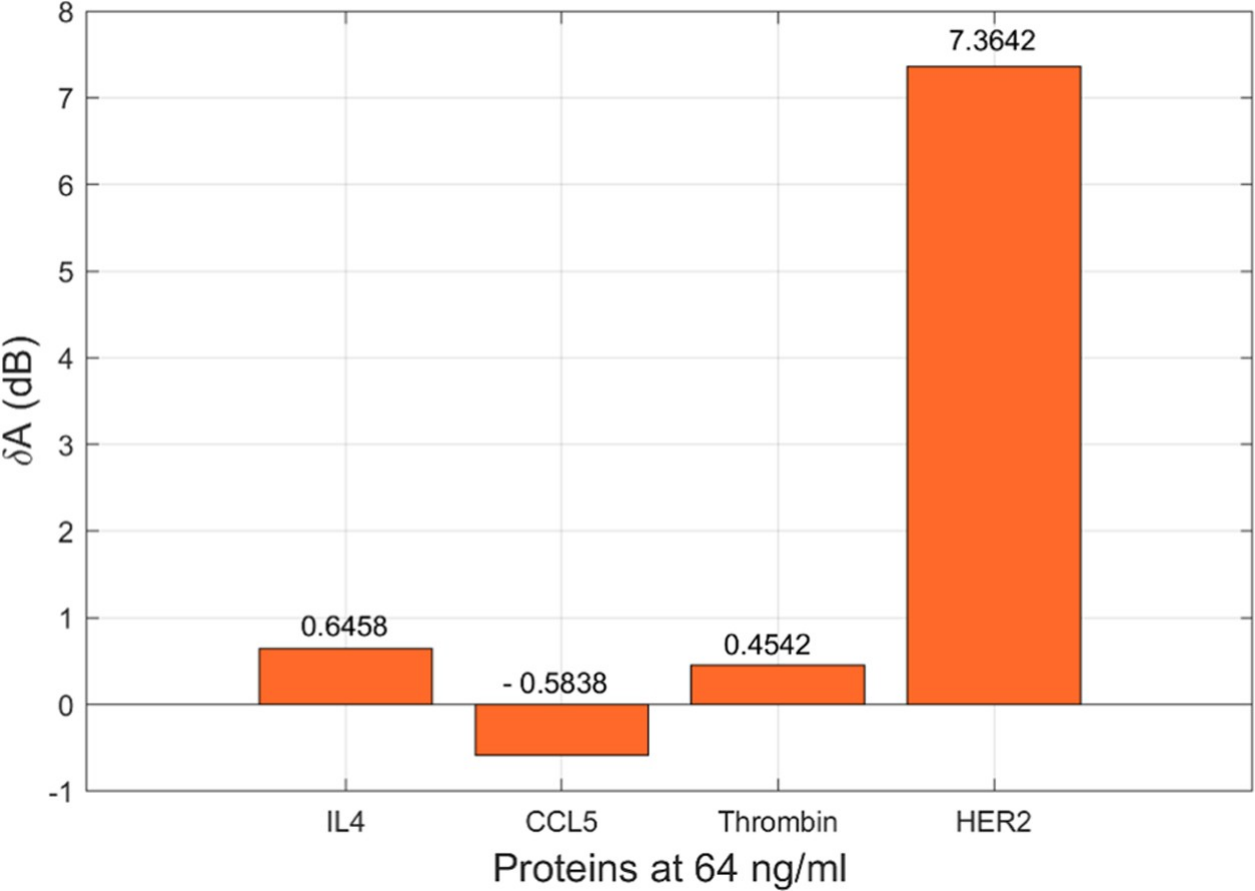
Specificity study of the TFBG–ball resonator sensor for the target sHER2 detection along with IL4, CCL5, and thrombin detection at 64 ng/mL concentration.

To evaluate the specificity of the TFBG–ball resonator, the sensor was tested for binding to different control proteins such as CCL5, IL4, and thrombin. CCL5 is an 8 kDa chemokine protein, which plays a role in recruiting leukocytes into inflammatory sites. Interleukin 4 (IL4) is a 15 kDa cytokine protein that regulates diverse T- and B-cell responses including differentiation of naive T cells. Thrombin is a 37 kDa enzyme that converts fibrinogen into fibrin, which is an integral step in clot formation. In theory, these proteins should not bind to the sensor surface as they should not be recognized by Trastuzumab. sHER, as well as other control proteins, were incubated at 64 ng/mL and the measurement was conducted in PBS after rinsing with PBS. Modes between 1554.7 and 1555 nm were chosen for the selectivity studies. Figure 5 shows the comparison of the amplitude change differences of the sensor. It was observed that the response of the sensor to sHER2 was 7.36 dB, whereas the response to other proteins was below 0.7 dB, indicating the specificity of the sensor for sHER.

From a device fabrication and performance standpoint, the TFBG–ball resonator device allows us to achieve excellent sensitivity, higher than that of most plasmonic TFBGs (a sevenfold sensitivity increase), without the need for additional elements such as polarizers or reflective mirrors on the fiber tip. The method for the fabrication of the fiber-tip sphere is fast and repeatable, and we aim at improving the integration of these two elements. Using an OBR device or another interrogator capable of resolving weakly reflective spectra, we can combine the excellent sensitivity of the device (over 4000 dB/RIU) with a detector having excellent power and wavelength resolution. In addition, the TFBG–ball resonator combines the spectral comb excited by the TFBG, which has high fringe visibility, with the profile of the ball resonator, which has an intrinsically higher RI sensitivity, such that each cladding mode has a narrow bandwidth and high sensitivity simultaneously. Thanks to these promising features, we could achieve a very low LoD, down to 151.5 ag/mL.

## 4 Conclusions

In this work, we reported the use of TFBGs together with ball resonators to create a sensitive device as compared to other optical-fiber-based sensors. The device was first tested in different sucrose solutions with varying RIs to establish its sensitivity (4034 dB/RIU). The sensitivity was obtained by tracking the most sensitive mode of the spectrum. The surface of the TFBG–ball resonator served as a platform for immobilizing the sHER2-specific antibody Trastuzumab and evaluating its performance for the detection of the breast cancer biomarker HER2. The immobilization of the antibody was achieved by silane coupling surface chemistry. The sensor exhibited a concentration-dependent response to increasing concentrations of sHER2 from 3 ag/mL to 128 ng/mL, with the lowest experimental detection threshold reported so far for sHER2. In addition to their small size and increased sensitivity, the fabrication of the TFBG–ball resonator sensors was relatively easy and robust. The small size of the sensor would also allow us to further incorporate it within microfluidic devices to minimize the sample volume as well as to develop a multiplexed setup for the simultaneous detection of several analytes. The current sensor is able to detect sHER2 in both buffer and serum with an LoD of 151.5 ag/mL and 3.7 pg/mL, respectively. The exceedingly low sHER2 limit of detection of this conceptually novel biosensor is encouraging; it is now conceivable to envisage the lowering of the diagnostic threshold for the early detection of breast cancer and its relapse.

## Notes

M.S. was responsible for conceptualization, investigation, methodology, validation, writing the original draft, and reviewing and editing it. A.A. was responsible for data curation, formal analysis, software, and methodology. Á.G.-V. performed sensor fabrication and writing, reviewing, and editing the manuscript. C.C. was responsible for supervision and writing, reviewing, and editing the manuscript. L.V. was responsible for conceptualization, supervision, funding acquisition, and writing, reviewing, and editing the manuscript. D.T. was responsible for conceptualization, supervision, funding acquisition, formal analysis, and writing, reviewing, and editing the manuscript.
